# Carbapenemase genes distribution in clonal lineages of *Acinetobacter baumannii*: a comprehensive study on plasmids and chromosomes

**DOI:** 10.3389/fcimb.2023.1283583

**Published:** 2023-12-01

**Authors:** Masoumeh Beig, Farzad Badmasti, Hamid Solgi, Vajihe Sadat Nikbin, Mohammad Sholeh

**Affiliations:** ^1^ Department of Bacteriology, Pasteur Institute of Iran, Tehran, Iran; ^2^ Isfahan Endocrine and Metabolism Research Center, Isfahan University of Medical Sciences, Isfahan, Iran

**Keywords:** *Acinetobacter baumannii*, carbapenem resistant, carbapenemase, gene repetition, sequence type

## Abstract

**Background:**

The global spread of plasmids carrying carbapenemase genes within carbapenem resistant *Acinetobacter baumannii* (CRAB) strains poses a worldwide public health issue. In this study, we conducted a comprehensive genetic analysis of plasmids and chromosomes harboring the major carbapenemase genes (*bla*_NDM_, *bla*_KPC_, *bla*_VIM_, *bla*_IMP_, *bla*_GES_, *bla*_OXA-58_-like, *bla*_OXA-24/40_-like, *bla*_OXA-143_-like, and *bla*_OXA-23_-like) in CRAB strains using bioinformatic tools.

**Methods:**

We retrieved plasmids and chromosomes carrying the major carbapenemase genes from GenBank. The size, replicon type, and conjugal apparatus of the plasmids were also determined. Furthermore, allele types, co-existence of other antimicrobial resistance genes alongside carbapenemases in plasmids or chromosomes, co-occurrence of carbapenemase genes, gene repetition, and sequence types (ST) of whole genomes were characterized.

**Results:**

The database contained 113 plasmids and 38 chromosomes harboring carbapenemase genes. This investigation revealed that *bla*_NDM_ and *bla*_OXA-58_-like were the predominant allele types in both the plasmids and chromosomes. Nine (7.96%) plasmids with *bla*_NDM-1_ were potentially conjugative. The most common replicon types of the plasmids were R3-T1, R3-T8, R3-T2, R3-T23, and RP-T1. The analysis revealed that *bla*_NDM-1_ and *bla*_OXA-58_-like genes possessed the highest variety of co-existence with other antibiotic resistance genes. The co-occurrence of dual carbapenemases was identified in 12 plasmids and 19 chromosomes. Carbapenemase gene repetitions were identified in 10 plasmids and one chromosome. Circular alignment revealed that the plasmids carrying the co-occurrence of *bla*_NDM-1_ and *bla*_OXA-58_ were more homogeneous. However, there was heterogeneity in certain regions of these plasmids. According to the minimum spanning tree (MST) results, the majority of the plasmids belonged to the genomes of ST2^Pas^, ST1^Pas^, ST422^Pas^, ST622^Pas^, and ST85^Pas^.

**Conclusion:**

*A. baumannii* appears to have a strong ability for genome plasticity to incorporate carbapenemase genes on its plasmids and chromosomes to develop resistance against carbapenems. Mobilizable plasmids harboring carbapenemases significantly contribute to the dissemination of these genes. The genetic structure of the plasmids revealed a strong associations of class I integrons, IS*Aba*-like structures, Tn*4401* elements, and *aac (6′)-Ib* with carbapenemases. Furthermore, gene repetition may also be associated with carbapenem heteroresistance.

## 1 Introduction

*Acinetobacter baumannii* is a member of the ESKAPE group (*Enterococcus faecium*, *Staphylococcus aureus*, *Klebsiella pneumoniae*, *A. baumannii*, *Pseudomonas aeruginosa*, and *Enterobacter* spp.). *A. baumannii* is an opportunistic pathogen that has been identified as an important cause of nosocomial infections, including ventilator-associated pneumonia in intensive care units, urinary tract, and bloodstream infections (Flores-Paredes et al., 2021; Nocera et al., 2021). These infections are associated with significant mortality rates owing to high levels of antimicrobial resistance (Alrahmany et al., 2022). Carbapenems are used as a last resort to treat severe infections caused by multidrug-resistant (MDR) and extensively drug-resistant (XDR) strains (Zalegh et al., 2023). In addition, carbapenem-resistant *A. baumannii* (CRAB) isolates have been categorized by the World Health Organization (WHO) as one of the 12 top priority resistant bacteria that pose the greatest threat to public health (Hsieh et al., 2020). The main cause of carbapenem resistance in *A. baumannii* isolates is the acquisition of metallo-beta-lactamases (MBLs), class A carbapenemases (for example, KPC), and carbapenem-hydrolyzing class D beta-lactamase (CHDLs) enzymes (Kyriakidis et al., 2021).

Treatment of antibiotic-resistant *A. baumannii* isolates is becoming increasingly difficult due to the co-existence of carbapenemase-encoding and other antibiotic-resistance genes, including aminoglycoside-modifying genes and plasmid-mediated quinolone resistance (Wasfi et al., 2021). However, these resistance genes are often located in mobile genetic elements (MGEs), including conjugative plasmids, transposons, integrons, and insertion sequences. They can be easily expanded and transferred between various bacterial species (Horne et al., 2023). Conjugative plasmids, which include the origin of the transfer (*oriT*) region, relaxase enzyme, type IV coupling protein (T4CP), and a gene cluster for the bacterial type IV secretion system (T4SS) apparatus, are essential for the horizontal gene transfer and dissemination of carbapenemase genes (Li et al., 2018). Lam et al. (2022) classified *A. baumannii* plasmids into three categories based on the Pfam domains of the replication initiation protein including RP, R1, and R3. Plasmids that contain conjugation genes and are often associated with various acquired antibiotic resistance genes belong to the four most common types: RP-T1, R3-T1, R3-T2, and R3-T3 (Lam et al., 2023). Plasmids carrying resistance genes are strongly related to specific sequence types (STs), including ST2, ST1, and ST3, which can cause nosocomial infections and outbreaks (Frenk et al., 2022). Therefore, the genetic characterization of these plasmids in successful international clones is important for understanding the carbapenemase-harboring bacteria and for effective decision-making in infection prevention strategies.

In this study, we conducted an *in silico* analysis and comparative assessment of carbapenemase harboring plasmids (CHPs) and chromosomes (CHCs) in *A. baumannii*. The primary objective was to identify the genetic structures of various allele types of the major carbapenemase genes (*bla*_IMP_, *bla*_VIM_, *bla*_GES_, *bla*_NDM_, *bla*_OXA-58-_like, *bla*_OXA24/40_-like, *bla*_OXA-143_-like, and *bla*_OXA-23_-like) using bioinformatic tools. Additionally, this study characterized the genetic properties of both CHPs and CHCs, encompassing replicon types, conjugation ability, co-existence (linkage of carbapenemases with other antimicrobial resistance genes), co-occurrence (presence of at least two carbapenemase genes in one strain), gene repetition, and phylogenetic relatedness.

## 2 Materials and methods

### 2.1 Preparation of the initial dataset and characterization of carbapenemases

Nucleotide sequences of nine major carbapenemase genes (*bla*_NDM_, *bla*_KPC_, *bla*_VIM_, *bla*_IMP_, *bla*_GES_, *bla*_OXA-58-_like, *bla*_OXA-24/40_-like, *bla*_OXA-143_-like, and *bla*_OXA-23_-like) in *A. baumannii* strains were retrieved from GenBank (https://www.ncbi.nlm.nih.gov/genbank/) (Benson et al., 2017). Two BLAST approaches including Microbial BLAST (https://blast.ncbi.nlm.nih.gov/Blast.cgi?PAGE_TYPE=BlastSearch&BLAST_SPEC=MicrobialGenomes) and Standard Nucleotide BLAST (https://blast.ncbi.nlm.nih.gov/Blast.cgi?PROGRAM=blastn&BLwwereeee_SPEC=GeoBlast&PAGcompleteSearch) was used to retrieve all completed plasmids and partial DNA fragments carrying carbapenemase genes, respectively. The criteria used to retrieve all carbapenemases were ≥ 80% identity and 100% coverage.

### 2.2 Allele types and genetic environment of carbapenemases

The allele types of the mentioned carbapenemase genes were determined (cut-off was 100% identity and 100% coverage) using the beta-lactamase database (http://bldb.eu/) (Naas et al., 2017). Additionally, the prevalence of each allele type was determined. Moreover, the genetic contexts of nine carbapenemase genes present on both plasmids and chromosomes were characterized. The most prevalent of genetic structure for each gene was depicted.

### 2.3 Detection of other AMR genes on the retrieved DNAs

The Comprehensive Antibiotic Resistance Database (CARD) (https://card.mcmaster.ca/home) was used to identify the presence of various antimicrobial resistance genes against beta-lactams, carbapenems, macrolides, aminoglycosides, and other antibiotics (Islam et al., 2023). The co-existence (gene linkage) of *A. baumannii* plasmids and chromosomes harboring major carbapenemase genes with various antimicrobial resistance-associated genes was investigated. Co-occurrence was determined based on the presence of at least two major carbapenemase genes in an isolate.

### 2.4 Genetic characterization and replicon typing of plasmids carrying carbapenemases

The conjugation apparatus of the plasmids was identified using oriTfinder (https://bioinfo-mml.sjtu.edu.cn/oriTfinder/) (Li et al., 2018). The presence or absence of conjugation elements, including *oriT*, relaxase-encoding genes, T4CP, and T4SS in the CHPs was determined using this web tool. The replicon type for each plasmid was determined using local BLASTn. In addition, carbapenemase gene repetition in the plasmids was performed. Moreover, information on the geographical regions, isolation sources, collection date, host disease, and hosts of all isolates harboring crabapenemase genes were extracted.

### 2.5 Phylogenetic analysis and circular alignment of carbapenemase-harboring plasmids

We conducted a distribution analysis of bacterial accessory genomic elements using ClustAGE (https://bmcbioinformatics.biomedcentral.com) (Ozer, 2018). This tool creates a presence/absence matrix, which is subsequently converted into an unweighted pair-group method with arithmetic mean (UPGMA) dendrogram using the Anvi’o tool. UPGMA was used to determine the phylogenetic relationships among all CHPs (Xu et al., 2023). The results were visualized using the Interactive Tree Of Life (iTOL) platform (https://itol.embl.de) (Letunic and Bork, 2021). In addition, multiple circular alignments were applied to compare the similarity and heterogeneity of plasmids carrying *bla*_NDM-1_/*bla*_OXA58_-like using the BLAST Ring Image Generator (BRIG) version 0.95 (https://brig.sourceforge.net/) (Yao et al., 2022). Differences in the gene content of these plasmids were determined through pan-genome analysis using Roary (Rapid large-scale prokaryote pan-genome analysis) version 3.11.2 (http://sanger-pathogens.github.io/Roary) (Page et al., 2015), which operates on annotated assemblies in GFF3 format produced by Prokka (rapid prokaryotic genome annotation) version 1.14.5 (http://www.vicbioinformatics.com/software.prokka.shtml) (Seemann, 2014).

### 2.6 Clonal relatedness of strains harboring carbapenemase genes

The distribution of major carbapenemase genes among the different STs was assessed. For each plasmid, ST of the related chromosome was determined using seven housekeeping genes (*cpn60*, *fusA*, *gltA*, *pyrG*, *recA*, *rplB*, and *rpoB*) from the PubMLST database (https://pubmlst.org). The clonal relatedness of STs was characterized using PHYLOViZ version 2.0 (http://www.phyloviz.net) to generate a minimum spanning tree (MST) for all STs (Nascimento et al., 2017).

## 3 Results

### 3.1 The prevalence and the allele types of the various carbapenemase genes

**Figure 1:**
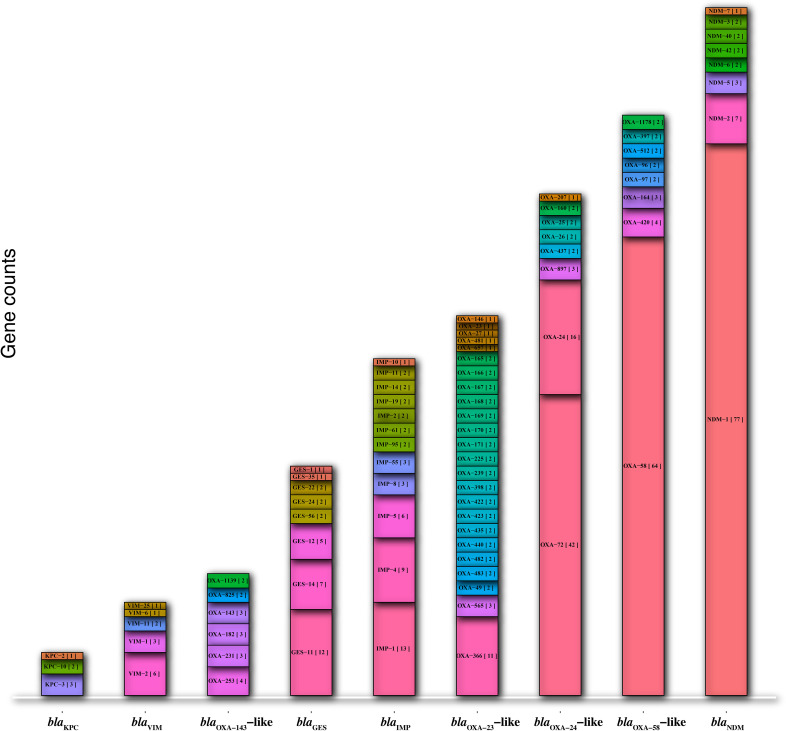
The prevalence of allele types in the nine major carbapenemase genes (*bla*_NDM_, *bla*_KPC_, *bla*_VIM_, *bla*_IMP_, *bla*_GES_, *bla*_OXA-58_-like, *bla*_OXA-24/40_-like, *bla*_OXA-143_-like, and *bla*_OXA-23_-like).

A total of 415 DNA fragments containing partial DNA plasmids (n= 264), complete plasmids (n= 113), and chromosomes (n= 38) carrying carbapenemase genes were extracted from the GenBank database using microbial and standard BLAST. The most prevalent alleles of carbapenemase genes found on the all retrieved DNAs were *bla*_NDM-1_ (77/96), *bla*_OXA-58_ (64/81), *bla*_OXA-72_ (42/70), *bla*_OXA-366_ (11/53), *bla*_IMP-1_ (13/47), *bla*_GES-11_ (12/32), *bla*_OXA-253_ (4/17), *bla*_VIM-2_ (6/13), and *bla*_KPC-3_ (3/6) (**Figure 1**).

### 3.2 The prevalence of carbapenemase genes carrying on plasmids and chromosomes

The prevalence of plasmids harboring carbapenemase genes was as follows: *bla*_OXA-24_ (46/113), *bla*_OXA-58_ (34/113), *bla*_NDM_ (25/113), and *bla*_GES_ (8/113). In contrast, the prevalence of chromosomes bearing these genes included *bla*_NDM_ (32/38). The detailed information is as follows: 12 *bla*_NDM-1_, one *bla*_NDM-2_, one *bla*_NDM-6_, one *bla*_NDM-1_/*bla*_OXA-58_-like, 17 *bla*_NDM-1_/*bla*_OXA-23_-like, *bla*_OXA-23_-like (3/38), *bla*_OXA-58_-like (2/38), and *bla*_KPC-3_ (1/38) (**Tables 1**, **2**).

**Table 1:** The data on the geographical regions, isolation sources, years, hosts, genetic characteristics and Rep types of the plasmids harboring carbapenemases isolated from *A. baumannii* strains.

Accession number	Plasmid	Geographic location	BioSample accession	Strain	Collection date	Host	Host disease	Isolation source	Molecular size (bp)	Gene	Rep*	ST*	Conjugal apparatus			
													*oriT*	Relaxase	T4SS	T4CP
NZ_CP008707.1	p1AB5075	USA	SAMN02894434	AB5075-UW	2008	Homo sapiens	Nosocomial infection	Osteomyelitis	83610	*bla*_GES_	RP-T1	1	–	+	+	–
NZ_KY022424.1	pAb8098	Tunisia	SAMN14227393	Ab8098	N.D	N.D	N.D	N.D	82667	*bla*_GES_	RP-T1	N.D	–	+	+	–
NZ_CP070361.1	pAB5075-VUB-itrA_3	Belgium	SAMN17898088	AB5075-VUB-itrA	2008	Homo sapiens	N.D	N.D	87892	*bla*_GES_	RP-T1	N.D	–	+	+	–
NZ_CP102817.1	unnamed3	Belgium	SAMN25131638	AB32-VUB	2014	Homo sapiens	N.D	N.D	88562	*bla*_GES_	RP-T1	158	–	+	+	–
NZ_CP070365.1	pAB5075-VUB_3	Belgium	SAMN17898087	AB5075-VUB	2008	Homo sapiens	N.D	N.D	83362	*bla*_GES_	RP-T1	1	–	+	+	–
NZ_LT984690.1	I	Kuwait	SAMEA104446236	K50	2008	N.D	N.D	N.D	79598	*bla*_GES_+ *bla*_OXA-23_-like	RP-T1	158	–	+	+	–
NZ_CP087299.1	p1OC070	Germany	SAMN23078463	OC070	N.D	Homo sapiens	N.D	N.D	83611	*bla*_GES_	RP-T1	400	–	+	+	–
CP087323.1	p2OC043	N.D	N.D	N.D	N.D	N.D	N.D	N.D	15932	*bla*_OXA-24_-like	R3-T1	2	–	–	–	–
MN266872.1	pAC1-BRL	N.D	N.D	N.D	N.D	N.D	N.D	N.D	16673	*bla*_OXA-24_-like	R3-T1	N.D		–	–	–
NZ_CP087311.1	p2OC068	Germany	SAMN23078460	OC068	N.D	Homo sapiens	N.D	N.D	83611	*bla*_GES_	RP-T1	85	–	+	+	–
GQ904227.1	pMMCU3	N.D	SAMN14225470	N.D	N.D	N.D	N.D	N.D	8964	*bla*_OXA-24_-like	R3-T2	N.D	–	–	–	–
KC503911.1	pNDM-AB	China	SAMN14225924	GF216	N.D	pig	N.D	Lung	47098	*bla*_NDM-1_	R3-T23	N.D	+	+	+	+
GQ377752.1	pMMA2	N.D	SAMN14225395	N.D	N.D	N.D	N.D	N.D	10679	*bla*_OXA-24_-like	R3-T2	N.D	–	–	–	–
GQ476987.1	pMMCU2	N.D	SAMN14225384	CU2	N.D	N.D	N.D	N.D	10270	*bla*_OXA-24_-like	R3-T8	N.D	–	–	–	–
CP048828.1	pABF9692	China	SAMN14072279	ABF9692	2017	Duck	N.D	Trachea	264805	*bla*_NDM-1_	R3-T17	23	–	–	+	–
CP047975.1	pDETAB2	China	SAMN13884837	DETAB-P2	2019	Homo sapiens	Cholecystitis	Rectal swab	100072	*bla*_NDM-1_	R3-T28	138	+	+	+	+
CP046901.1	pA1429a	China	SAMN13565236	A1429	2010	Homo sapiens	Nosocomial infection	Secretion	7852	*bla*_OXA-24_-like	R3-T8	108	–	–	–	–
CP043954.1	pK09-14	Malaysia	SAMN12769618	K09-14	2017	N.D	N.D	Soil	7791	*bla*_OXA-24_-like	R3-T24	46	–	–	–	–
CP042557.1	pE47_001	Australia	SAMN12289292	E47	2013	N.D	N.D	Room	327867	*bla*_OXA-58_-like*+ bla*_IMP_	N.D	1547	–	–	+	+
GQ904226.1	Pmmd	N.D	SAMN14225471	N.D	N.D	N.D	N.D	N.D	9964	*bla*_OXA-24_-like	R3-T8	N.D	–	–	–	–
EU294228.1	pABIR	N.D	SAMN14225727	trans conjugant 1 (MAD)	N.D	N.D	N.D	N.D	29823	*bla*_OXA-58_-like	R3-T8	N.D	–	–	–	–
CP031446.1	pAB120	USA	SAMN09769497	MDR-UNC	2012	Homo sapiens	Necrotizing fasciitis	ulcer	10879	*bla*_OXA-24_-like	R3-T1	2	–	–	–	–
CP087322.1	p1OC043	N.D	N.D	N.D	N.D	N.D	N.D	N.D	21542	*bla*_OXA-24_-like	R3-T1	2	–	–	–	–
JX069966.1	Pab120	Lithuania	SAMN14226317	K60	2010	Homo sapiens	N.D	Respiratory	10879	*bla*_OXA-24_-like	R3-T1	N.D	–	–	–	–
CP087318.1	p2OC064	N.D	N.D	N.D	N.D	N.D	N.D	N.D	37306	*bla*_OXA-58_-like	R3-T1	1	–	–	–	–
KT852971.1	p255n_1	Vietnam	SAMN14226423	255_n	2005	N.D	N.D	Nasal	92939	*bla*_OXA-58_-like	R3-T7	N.D	–	–	–	–
KR535993.1	pA105-2	Sweden	SAMN14226626	A105	2013	N.D	N.D	Bronchial	9830	*bla*_OXA-24_-like	R3-T1	N.D	–	–	–	–
CP104353.1	unnamed2	N.D	N.D	N.D	N.D	N.D	N.D	N.D	9935	*bla*_OXA-24_-like	R3-T8	422	–	–	–	–
CP071920.1	pABT-897-17	N.D	N.D	N.D	N.D	N.D	N.D	N.D	13480	*bla*_OXA-24_-like	R3-T1	45	–	–	–	–
KT946773.1	pBAL_204	N.D	N.D	N.D	N.D	N.D	N.D	N.D	17003	*bla*_OXA-24_-like	N.D	N.D	–	–	–	–
KT022421.1	pAB-ML	N.D	SAMN14226656	ML	N.D	N.D	N.D	N.D	12056	*bla*_OXA-24_-like	R3-T8	N.D	–	–	–	–
CP000864.1	Pacicu1	N.D	N.D	N.D	N.D	N.D	N.D	N.D	28279	*bla*_OXA-58_-like	R3-T1	2	–	–	–	–
KJ534569.1	AbATCC329	N.D	SAMN14226772	ATCC	N.D	N.D	N.D	N.D	8842	*bla*_OXA-24_-like	R3-T2	N.D	–	–	–	–
KJ534568.1	AbATCC223	N.D	SAMN14226773	ATCC	N.D	N.D	N.D	N.D	8840	*bla*_OXA-24_-like	R3-T2	N.D	–	–	–	–
KX230793.1	pMAL-1	N.D	SAMN14227146	MAL	N.D	N.D	N.D	Urine	9810	*bla*_OXA-24_-like	R3-T1	N.D	–	–	–	–
CP104344.1	unnamed2	N.D	N.D	N.D	N.D	N.D	N.D	N.D	9935	*bla*_OXA-24_-like	R3-T8	422		–	–	–
KY202458.1	pIBAC_oxa58_20C15	N.D	SAMN14227359	AB20C15	N.D	N.D	N.D	N.D	26781	*bla*_OXA-58_-like	R3-T1	N.D	–	+	–	–
KY984046.1	pAb242_12	N.D	N.D	N.D	N.D	N.D	N.D	N.D	11891	*bla*_OXA-58_-like	R3-T68	N.D	–	–	–	–
AP023079.1	pOCU_Ac16a_2	N.D	N.D	N.D	N.D	N.D	N.D	N.D	41087	*bla*_NDM-1_	R3-T23	412	+	+	+	+
KY984047.1	pAb242_2	Argentina	SAMN07509424	Ab242	1997	Homo sapiens	Nosocomial infection	Ascitic fluid	24808	*bla*_OXA-58_-like	R3-T32	15	–	+	+	–
CP003848.1	pBJAB0715	N.D	SAMN02604245	BJAB0715	N.D	N.D	N.D	N.D	52268	*bla*_OXA-58_-like	N.D	23	–	–	–	–
CP059730.1	pAbCTX13_7kb	N.D	N.D	N.D	N.D	N.D	N.D	N.D	7055	*bla*_OXA-24_-like	R3-T10	78	–	–	–	–
LN833432.1	pNDM-32	India	SAMEA3298506	N.D	N.D	N.D	N.D	Clinical	84623	*bla*_NDM-1_	R3-T17	N.D	–	–	–	–
CP107035.1	pABT-52Ts19	N.D	N.D	N.D	N.D	N.D	N.D	N.D	13953	*bla*_OXA-24_-like	R3-T1	2	–	–	–	–
JN377410.2	pAbNDM-1	N.D	N.D	N.D	N.D	N.D	N.D	N.D	48368	*bla*_NDM-1_	R3-T23	639	+	+	+	+
CP027184.1	unnamed2	N.D	SAMN04014893	N.D	N.D	N.D	N.D	N.D	60699	*bla*_OXA-58_-like	R3-T1	32	–	–	–	–
MN495625.1	pA2485	N.D	N.D	N.D	N.D	N.D	N.D	N.D	15405	*bla*_OXA-24_-like	R3-T2	N.D	–	–	–	–
CP027179.1	unnamed1	N.D	SAMN04014911	N.D	N.D	N.D	N.D	N.D	33036	*bla*_OXA-58_-like	R3-T1	32	–	–	–	–
CP027532.1	unnamed2	N.D	SAMN04014929	AR_0088	N.D	N.D	N.D	N.D	41087	*bla*_NDM-1_	R3-T23	25	+	+	+	+
MN495626.1	pA2503	N.D	N.D	N.D	N.D	N.D	N.D	N.D	15405	*bla*_OXA-24_-like	R3-T2	N.D	–	–	–	–
CP015365.1	pAba3207a	Mexico	SAMN04485290	3207	2008	Homo sapiens	N.D	Bronchial	13478	*bla*_OXA-58_-like	R3-T1	422	–	–	–	–
AP023080.1	pOCU_Ac16a_3	N.D	SAMD00059694	OCU_Ac16a	2015	N.D	N.D	N.D	13096	*bla*_OXA-58_-like	R3-T5	412	–	–	–	–
CP026127.1	pNDM-0285	USA	SAMN06040401	ABNIH28	2016	N.D	N.D	N.D	39359	*bla*_NDM-1_	R3-T23	1543	+	+	+	+
CP023032.1	pAba7847a	Mexico	SAMN07284119	7847	2008	Homo sapiens	Bacteremia	Blood	13478	*bla*_OXA-58_-like	R3-T17	1544	–	–	–	–
CP038646.1	unnamed2	India	SAMN11311113	ACN21	2018	Homo sapiens	Bacteremia	Blood	57333	*bla*_OXA-58_-like	R3-T60	85	–	–	–	–
MT002974.1	pAB17	N.D	N.D	N.D	N.D	N.D	N.D	N.D	41087	*bla*_NDM-1_	R3-T42	1230	+	+	+	+
CP023035.1	pAba5845a	Mexico	SAMN07520231	5845	2009	Homo sapiens	Nosocomial infection	Wound	9935	*bla*_OXA-24_-like	R3-T8	2	–	–	–	–
CP023027.1	pAba10042a	Mexico	SAMN07520233	10042	2011	Homo sapiens	Nosocomial infection	Secretion	10062	*bla*_OXA-24_-like	R3-T8	2	–	–	–	–
CP023021.1	pAba9201a	Mexico	SAMN07520236	9201	2013	Homo sapiens	Nosocomial infection	Blood	9024	*bla*_OXA-24_-like	R3-T8	422	–	–	–	–
CP024419.1	pA388	Greece	SAMN07736509	A388	2002	Homo sapiens	N.D	N.D	33036	*bla*_OXA-58_-like	R3-T19	1	–	–	–	–
CP027245.2	pOXA58_005078	China	SAMN08364584	WCHAB005078	N.D	Homo sapiens	N.D	N.D	70509	*bla*_OXA-58_-like	R3-T7	20	–	–	–	–
CP026749.2	pOXA58_005133	China	SAMN08364585	WCHAB005133	N.D	Homo sapiens	N.D	N.D	42455	*bla*_OXA-58_-like	R3-T21	2	–	–	–	–
CP031381.2	pACICU1b	Italy	SAMN09302593	ACICU	2005	Homo sapiens	Bacteremia	cerebrospinal	24268	*bla*_OXA-58_-like	R3-T1	2	–	–	–	–
CP030109.1	pDA33382-85	Germany	SAMN09460321	DA33382	N.D	Homo sapiens	N.D	Tracheal	84678	*bla*_OXA-58_-like	R3-T1	1	–	+	+	–
KY704308.1	pAbIHIT32296	Luxembourg	SAMN14227859	IHIT32296	2016	domestic	N.D	Nose	8493	*bla*_OXA-24_-like	R3-T24	294	–	–	–	–
CP038501.1	unnamed1	India	SAMN11298775	CIAT758	2018	Homo sapiens	Bacteremia	Blood	78125	*bla*_OXA-58_-like	R3-T2	10	–	–	–	–
CP087346.1	P2db007	Germany	SAMN23078449	DB007	N.D	Homo sapiens	N.D	N.D	11084	*bla*_OXA-24_-like	R3-T1	636	–	–	–	–
CP104348.1	unnamed2	USA	SAMN21493860	2021CK-01333	2021	Homo sapiens	N.D	sputum	335558	*bla*_NDM-1_ *+ bla*_OXA-58_	N.D	422	–	–	–	–
CP104352.1	unnamed1	USA	SAMN21493862	2021CK-01335	2021	Homo sapiens	N.D	Wound Abscess	335718	*bla*_NDM-1_ *+ bla*_OXA-58_	N.D	422	–	–	–	–
CP104447.1	unnamed2	USA	SAMN21924069	2021CK-01407	2021	Homo sapiens	N.D	sputum	335722	*bla*_NDM-1_ *+ bla*_OXA-58_	N.D	N.D	–	–	–	–
CP104336.1	unnamed4	USA	SAMN21924070	2021CK-01408	2021	Homo sapiens	N.D	Blood	328511	*bla*_NDM-1_ *+ bla*_OXA-58_	N.D	N.D	–	–	–	–
CP104339.1	unnamed2	USA	SAMN21924071	2021CK-01409	2021	Homo sapiens	N.D	Wound Abscess	329791	*bla*_NDM-1_ *+ bla*_OXA-58_	N.D	422	–	–	–	–
CP087376.1	p2DB001	Germany	SAMN23078443	DB001	N.D	Homo sapiens	N.D	N.D	9830	*bla*_OXA-24_-like	R3-T1	636	–	–	–	–
CP087353.1	p2DB003	Germany	SAMN23078445	DB003	N.D	Homo sapiens	N.D	N.D	11084	*bla*_OXA-24_-like	R3-T1	422	–	–	–	–
CP087373.1	p2DB004	Germany	SAMN23078446	DB004	N.D	Homo sapiens	N.D	N.D	11084	*bla*_OXA-24_-like	R3-T1	636	–	–	–	–
CP087368.1	p4DB005	Germany	SAMN23078447	DB005	N.D	Homo sapiens	N.D	N.D	12457	*bla*_OXA-24_-like	R3-T1	636	–	–	–	–
CP104345.1	unnamed2	USA	SAMN21493859	2021CK-01332	2021	Homo sapiens	N.D	Wound Abscess	338291	*bla*_NDM-1_ *+ bla*_OXA-58_	N.D	422	–	–	–	–
CP087324.1	p3OC043	Germany	SAMN23078457	OC043	N.D	Homo sapiens	N.D	N.D	14741	*bla*_OXA-24_-like	R3-T1	2	–	–	–	–
CP087319.1	p1OC064	Germany	SAMN23078458	OC064	N.D	Homo sapiens	N.D	N.D	36555	*bla*_OXA-58_-like	R3-T1	1	–	–	–	–
CP102825.1	unnamed	Belgium	SAMN25131632	AB3-VUB	2017	Homo sapiens	N.D	N.D	12543	*bla*_OXA-58_-like	R3-T10	2	–	–	–	–
CP102814.1	unnamed1	Belgium	SAMN25131633	AB9-VUB	2014	Homo sapiens	N.D	N.D	9830	*bla*_OXA-24_-like	R3-T1	636	–	–	–	–
CP102818.1	unnamed1	Belgium	SAMN25131641	AB40-VUB	2014	Homo sapiens	N.D	N.D	15315	*bla*_OXA-24_-like	N.D	78	–	–	–	–
CP098792.1	p1VB280820	India	SAMN28980502	280820	2020	Homo sapiens	Bacteremia	Blood	141629	*bla*_NDM-1_ *+ bla*_OXA-58_	R3-T21	126	–	–	–	–
CP098796.1	p1VB280821	India	SAMN28981486	VB280821	2020	Homo sapiens	Bacteremia	Blood	141631	*bla*_NDM-1_ *+ bla*_OXA-58_	R3-T21	126	–	–	–	–
CP101888.1	Pccbh31258	Brazil	SAMN29977860	CCBH31258	2021	Homo sapiens	Nosocomial infection	catheter	340418	*bla*_NDM-1_	R3-T20	374	–	–	–	–
CP101886.1	pCCBH31270	Brazil	SAMN29981016	CCBH31270	2021	Homo sapiens	Nosocomial infection	tracheal	340708	*bla*_NDM-1_ *+ bla*_OXA-58_	N.D	374	–	–	–	–
CP102765.1	pAOR07BL-3	China	SAMN30248452	AOR07-BL	2020	Homo sapiens	Bacteremia	Blood	31683	*bla*_OXA-58_-like	R3-T33	40	–	+	+	–
OK492156.1	pABCTX2	N.D	N.D	N.D	N.D	N.D	N.D	N.D	15266	*bla*_OXA-24_-like	N.D	N.D	–	–	–	–
CP053220.1	unnamed2	Tanzania	SAMN14833556	DT01139C	2017	Homo sapiens	Bacteremia	N.D	63650	*bla*_NDM-1_	R3-T20	232	+	+	+	+
KY202456.1	pIBAC_oxa58_1433	N.D	SAMN14227361	AB1433	N.D	N.D	N.D	N.D	26496	*bla*_OXA-58_-like	R3-T1	N.D	–	+	–	–
MK053932.1	pIEC383	Brazil	SAMN14227958	IEC383	2014	N.D	N.D	Blood	47283	*bla*_NDM-1_	R3-T23	N.D	+	+	+	+
MG100202.1	pAb825_36	N.D	SAMN14228542	Ab825	N.D	N.D	N.D	Wound	35743	*bla*_OXA-58_-like	R3-T32	15	–	+	–	–
OK492158.1	pAbCTX17a	N.D	N.D	N.D	N.D	N.D	N.D	N.D	17003	*bla*_OXA-24_-like	N.D	N.D	–	–	–	–
OK492157.1	pAbCTX11	N.D	N.D	N.D	N.D	N.D	N.D	N.D	15343	*bla*_OXA-24_-like	N.D	N.D	–	–	–	–
OK546135.1	pAbCTX16	N.D	N.D	N.D	N.D	N.D	N.D	N.D	19656	*bla*_OXA-24_-like	R3-T1	78	–	–	–	–
MK323042.1	pAb45063_a	N.D	SAMN14228622	Acb-45063	N.D	N.D	N.D	N.D	19808	*bla*_OXA-58_-like	R3-T14	N.D	–	–	–	–
FM210331.1	Pabva01	Italy	SAMN14229501	VA-56600	2000	N.D	N.D	Broncho alveolar	8963	*bla*_OXA-24_-like	R3-T2	N.D	–	–	–	–
CP050416.1	pPM193665_1	India	SAMN14420254	PM193665	2019	Homo sapiens	Wound	Pus	150385	*bla*_NDM-1_	R3-T23	10	–	–	–	–
CP050426.1	pPM194122_1	India	SAMN14420255	PM194188	2019	Homo sapiens	Pneumonia	BAL	150385	*bla*_NDM-1_	R3-T23	10	–	–	–	–
CP051867.1	pAb-C63_1	Ghana	SAMN14667517	Ab-C63	2016	Homo sapiens	N.D	Sputum	81353	*bla*_OXA-58_-like	R3-T25	107	–	–	–	–
CP051863.1	pAb-C102_1	Ghana	SAMN14667518	Ab-C102	2016	Homo sapiens	N.D	Blood	90089	*bla*_OXA-58_-like	R3-T25	1472	–	–	–	–
KY202457.1	pIBAC_oxa58_2RED	N.D	SAMN14227360	AB2RED09	N.D	N.D	N.D	N.D	25311	*bla*_OXA-58_-like	R3-T1	N.D	–	+	–	–
CP059731.1	pAbCTX13_17kb	France	SAMN15501316	AbCTX13	2017	Homo sapiens	Peritoneal	Peritoneal	17003	*bla*_OXA-24_-like	N.D	78	–	–	–	–
CP087356.1	p2DB002	N.D	N.D	N.D	N.D	N.D	N.D	N.D	11084	*bla*_OXA-24_-like	R3-T1	636	–	–	–	–
CP059301.1	pAC1633	Malaysia	SAMN15574350	AC1633	2016	Homo sapiens	Pneumonia	Blood	174292	*bla*_NDM-1_	R3-T57	126	–	–	–	–
CP059478.1	p17-84_OXA	China	SAMN15637465	17-84	2017	Homo sapiens	N.D	N.D	108715	*bla*_OXA-58_-like	R3-T28	N.D	–	+	–	–
OK492155.1	pAbCTX19	N.D	N.D	N.D	N.D	N.D	N.D	N.D	8970	*bla*_OXA-24_-like	R3-T1	N.D	–	–	–	–
CP081138.1	pARLG_6420_1	USA	SAMN16351204	ARLG_6420	2018	Homo sapiens	N.D	Blood	11323	*bla*_OXA-24_-like	R3-T1	N.D	–	–	–	–
AB823544.1	pAB-NCGM253	Russia	SAMN18276099	GIMC5510	2017	Homo sapiens	Sepsis	Blood	8970	*bla*_OXA-24_-like	R3-T1	N.D	–	–	–	–
CP072528.1	pDETAB5	China	SAMN18498586	DETAB-E227	2019	N.D	N.D	N.D	97035	*bla*_OXA-58_-like	R3-T7	1554	–	–	–	–
CP076809.1	p2UC20804	Chile	SAMN19356540	UC20804	2010	Homo sapiens	Bacteremia	Peritoneal	17469	*bla*_OXA-58_-like	R3-T14	109	–	–	–	–
CP084298.1	pLHC22-2-tetX-162k	China	SAMN20477964	LHC22-2	2020	N.D	N.D	Feces	162264	*bla*_OXA-58_-like	R3-T21	2253	–	–	–	–
CP104343.1	unnamed1	USA	SAMN21493827	2021CK-01300	2021	Homo sapiens	N.D	Wound Abscess	335718	*bla*_NDM-1_ *+ bla*_OXA-58_	N.D	422	–	–	–	–

*Rep, replicase (rep) gene of plasmids.

*ST, Sequence type.

N.D, Not determined.

Presence (+), Absence (-).

**Table 2:** The data on the geographical regions, isolation sources, years, hosts, and clonal relatedness of the chromosomes of *A. baumannii* strains harboring carbapenemases.

Accession number	Geographic location	BioSample accession	Strain	Collection date	Host	Host disease	Isolation source	Gene	ST*	Molecular size (bp)
**CP038644.1**	India	SAMN11311113	ACN21	2018	Homo sapiens	Bacteremia	Blood	*bla*_NDM-1_	85	3827138
**CP053215.1**	Tanzania	SAMN14833494	DT0544C	2017	Homo sapiens	Bacteremia	N.D	*bla*_NDM-1_	374	3912346
**CP050403.1**	India	SAMN14414761	VB2486	2019	Homo sapiens	Pneumonia	Sputum	*bla*_NDM-1_	1	4137731
**CP050523.1**	India	SAMN14410111	VB7036	2019	Homo sapiens	Bacteremia	Blood	*bla*_NDM-1_ *+ bla*_OXA-23_	2	4052480
**CP050388.1**	India	SAMN14409628	VB473	2019	Homo sapiens	Respiratory	Sputum	*bla*_NDM-1_ *+ bla*_OXA-23_	2	3948250
**CP085788.1**	N.D	SAMN14226727	RCH52	N.D	N.D	N.D	N.D	*bla*_OXA_ *_-_*_565_ *+ bla*_OXA-10_	729	4023505
**CP040087.1**	India	SAMN11571817	VB35575	2018	Homo sapiens	Sepsis	Blood	*bla*_NDM-1_ *+ bla*_OXA-23_	2	4031418
**CP040050.1**	India	SAMN11557458	VB16141	2019	Homo sapiens	Sepsis	Blood	*bla*_NDM-1_ *+ bla*_OXA-23_	622	4082961
**CP034427.1**	India	SAMN11554995	VB1190	2019	Homo sapiens	Sepsis	Blood	*bla*_NDM-1_ *+ bla*_OXA-58_	1786	3240866
**CP040040.1**	India	SAMN11554497	VB958	2019	Homo sapiens	Sepsis	Blood	*bla*_NDM-1_ *+ bla*_OXA-23_	Unknown	2944648
**KJ748372.1**	Puerto Rico	SAMN03175026	M3AC9-7	2009	Homo sapiens	septicemia	Blood	*bla*_KPC-3_	250	97550
**CP035930.1**	India	SAMN10170272	VB31459	2017	Homo sapiens	Bacteremia	Blood	*bla*_NDM-1_	Unknown	3078300
**CP026338.1**	Mexico	SAMN07559626	810CP	2015	Homo sapiens	Acute Myeloid Leukemia	feces	*bla*_OXA-23_-like	156	4089681
**CP018861.2**	Mexico	SAMN06093832	11510	2012	Homo sapiens	N.D	Bronchial	*bla*_OXA-23_-like	156	4085913
**LN997846.1**	France	SAMEA3715145	N.D	2014	N.D	N.D	N.D	*bla*_NDM-1_	126	3939746
**CP027528.1**	N.D	SAMN04014924	AR_0083	N.D	N.D	N.D	N.D	*bla*_NDM-1_ *+ bla*_OXA-23_	1	4149444
**LN868200.1**	France	SAMEA3751109	N.D	2013	N.D	N.D	N.D	*bla*_NDM-1_	267	3819158
**CP021345.1**	India	SAMN03771550	B11911	2014	Homo sapiens	Bacteremia	Blood	*bla*_NDM-1_ *+ bla*_OXA-23_	149	4018724
**AP014649.1**	Nepal	SAMD00020223	IOMTU433	2013	Homo sapiens	N.D	N.D	*bla*_NDM-1_ *+ bla*_OXA-23_	622	4000970
**CP072290.1**	India	SAMN18452698	KSK18	2020	Homo sapiens	Respiratory	Respiratory	*bla*_NDM-1_ *+ bla*_OXA-23_	622	4095769
**CP091350.1**	Belgium	SAMN25131660	AB212-VUB	N.D	Homo sapiens	N.D	N.D	*bla*_OXA-58_	2	3922329
**CP091361.1**	Belgium	SAMN25131649	AB177-VUB	N.D	Homo sapiens	N.D	N.D	*bla*_NDM-1_	85	4014141
**CP088894.1**	China	SAMN23553143	XH1935	2021	Homo sapiens	N.D	Sputum	*bla*_NDM-1_ *+ bla*_OXA-23_	164	3858469
**CP088895.1**	China	SAMN23553142	DETAB-R21	2021	Homo sapiens	N.D	Rectal swab	*bla*_NDM-1_ *+ bla*_OXA-23_	164	3862196
**CP087312.1**	Germany	SAMN23078459	OC059	N.D	Homo sapiens	N.D	N.D	*bla*_NDM-2_	103	3860314
**CP086759.1**	Germany	SAMN23041240	ACI713	2018	Homo sapiens	N.D	N.D	*bla*_OXA-58_	2	3935674
**CP082952.1**	Lebanon	SAMN21240079	Cl300	2015	Homo sapiens	Respiratory	Tracheal aspirate	*bla*_NDM-1_	85	4007379
**CP072300.1**	India	SAMN18452712	KSK20	2020	Homo sapiens	Respiratory	Respiratory	*bla*_NDM-1_ *+ bla*_OXA-23_	622	4095769
**CP072295.1**	India	SAMN18452699	KSK19	2020	Homo sapiens	Respiratory	Respiratory	*bla*_NDM-1_ *+ bla*_OXA-23_	622	4095778
**CP040047.1**	Germany	SAMN25263435	Nord4-2	2018	Homo sapiens	N.D	N.D	*bla*_NDM-1_	Unknown	3294487
**CP072285.1**	India	SAMN18452659	KSK11	2020	Homo sapiens	Respiratory	Respiratory	*bla*_NDM-1_	622	4080146
**CP072280.1**	India	SAMN18452342	KSK10	2020	Homo sapiens	Respiratory	Respiratory	*bla*_NDM-1_+ *bla*_OXA-23_	622	4096957
**CP072275.1**	India	SAMN18452334	KSK7	2020	Homo sapiens	Respiratory	Respiratory	*bla*_NDM-1_+ *bla*_OXA-23_	622	4095769
**CP072270.1**	India	SAMN18452234	KSK6	2020	Homo sapiens	Respiratory	Respiratory	*bla*_NDM-1_+ *bla*_OXA-23_	622	4091200
**CP072398.1**	India	SAMN18451305	KSK2	2020	Homo sapiens	Respiratory	Respiratory	*bla*_NDM-1_	622	4081424
**CP072122.1**	India	SAMN18396008	KSK1	2020	Homo sapiens	Respiratory	Respiratory	*bla*_NDM-1_	622	4038785
**CP060013.1**	USA	SAMN15738014	TP3	2016	Homo sapiens	Nosocomial infection	Human clinical	*bla*_NDM-1_+ *bla*_OXA-23_	570	3871732
**CP065392.1**	Spain	SAMN15676490	AbBAS-1	2019	Homo sapiens	Dysuria	Clinical	*bla*_NDM-6_	85	4007838

*ST, Sequence type.

N.D, Not determined.

### 3.3 Genetic characterization of plasmids harboring carbapenemases

The size of the complete plasmids harboring carbapenemase genes varied from 83,610 to 335,718 bp. The majority of plasmids carrying carbapenemase genes, such as *bla*_NDM_, *bla*_OXA-58_-like, and *bla*_OXA-24_-like, fell within the range of 10,000 to 50,000 bp. In contrast, plasmids housing *bla*_GES_ genes tended to be larger, typically in the range of 50,000 to 100,000 bp. Nine (7.96%, 9/113) plasmids were identified as potentially conjugative, as they carried all four essential conjugal components including *oriT*, relaxase, type IV coupling protein (T4CP), and type IV secretion system (T4SS). The presence of *bla*_GES_ was exclusively confined to plasmids with RP-T1 replicon type (100%, 8/8). On the other hand, the *bla*_NDM_ genes were primarily related to plasmids with the R3-T23 replicon type (32%, 8/25). Similarly, *bla*_OXA-24_ and *bla*_OXA-58_ were predominantly associated of plasmids with the R3-T1 replicon type (45.65%, 21/46 and 32.35%, 11/34) (**Table 1**).

### 3.4 Epidemiological data on *A. baumannii* harboring carbapenemases in plasmids and chromosomes

In this study, a notable proportion of *A. baumannii* strains harboring carbapenemases in plasmids and chromosomes were derived from blood samples. Furthermore, it is important to highlight that all hosts of these isolates were *Homo sapiens*. Respiratory and nosocomial infections were the predominant diseases associated with the isolates. Notably, plasmids carrying the *bla*_GES_ (25%, 2/8), *bla*_OXA-24_-like (13.04%, 6/46), *bla*_NDM_ (32%, 8/25), and *bla*_OXA-58_-like (17.6%, 6/34) genes were identified in Germany, the United States, and China, respectively. Additionally, a substantial percentage of chromosomes harboring carbapenemases was identified in India (50%, 19/38). Detailed information on the geographical regions, isolation sources, collection date, host disease, and hosts of plasmids and chromosomes carrying carbapenemases is shown in **Tables 1**, **2**.

### 3.5 The co-existence of other antimicrobial resistance genes in the plasmids and chromosomes harboring carbapenemases

**Figure 2:**
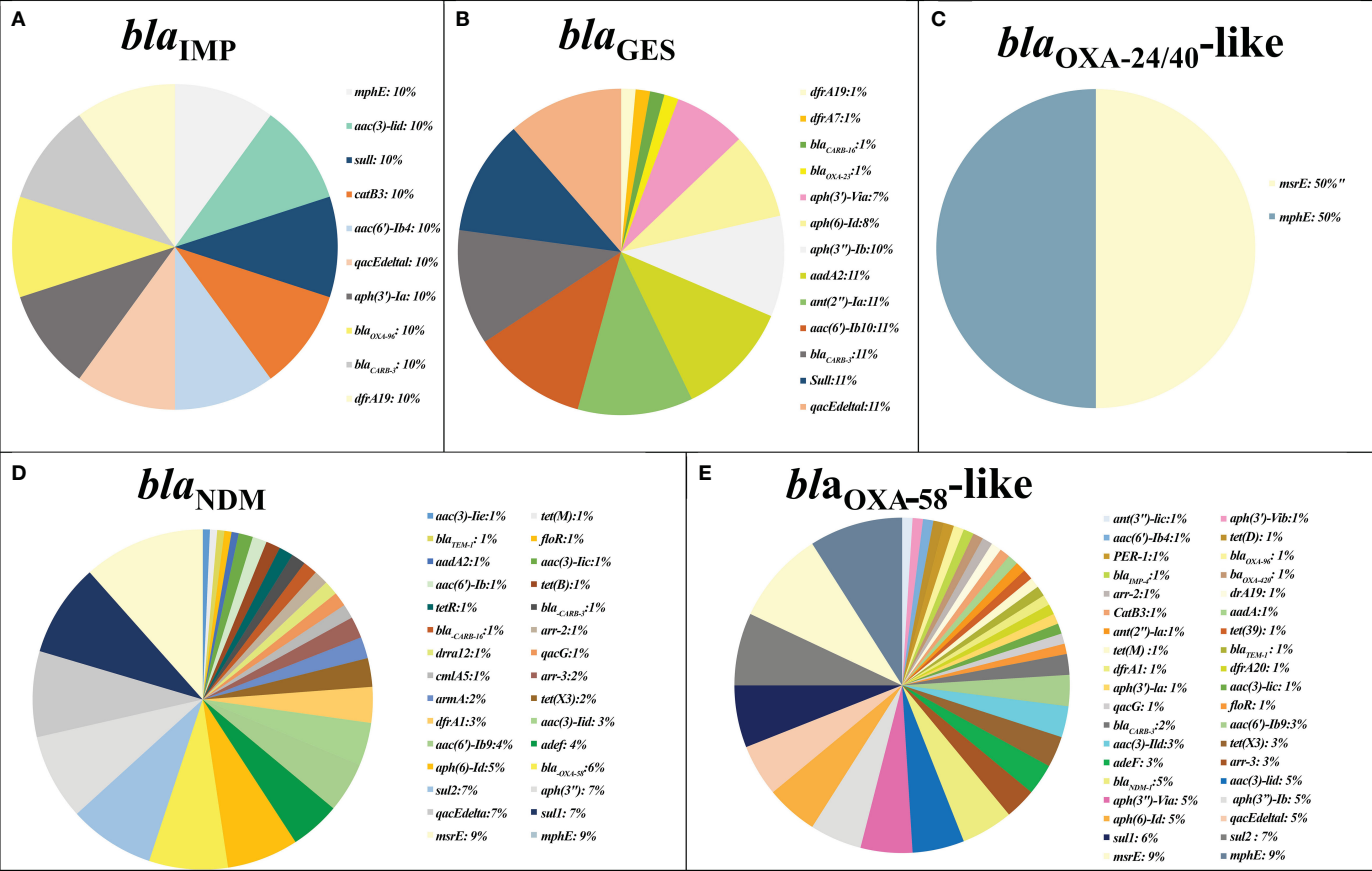
The prevalence of other antimicrobial resistance in plasmids containing major carbapenemase genes. The co-existence rate of other antibiotic resistance genes detected in plasmids harboring *bla*_IMP_ gene **(A)**, *bla*_GES_ gene **(B)**, *bla*_OXA-24/40_-like gene **(C)**, *bla*_NDM_ gene **(D)**, and *bla*_OXA-58_-like **(E)**.

Antimicrobial resistance genes against various classes of antibiotics, including beta-lactams, carbapenems, macrolides, aminoglycosides, sulfonamides, trimethoprim (TMP), tetracyclines, rifampin, fluoroquinolones, and phenicols, along with genes encoding efflux pump proteins and antiseptic resistance genes, were found in plasmids and chromosomes carrying carbapenemase genes. Carbapenemase gene carriage is a prerequisite for plasmid selection. Co-existence of *bla*_IMP_ and resistance genes against macrolides, aminoglycosides, beta-lactams, carbapenems, sulfonamides, TMP, phenicols, and a disinfectant (*qacEdelta1*) was observed. Additionally, *bla*_GES_ co-existed with resistance genes against aminoglycosides, beta-lactams, carbapenems, sulfonamides, TMP, and a disinfectant (*qacEdelta1*). The *bla*_OXA-24/40_-like-resistant genes related to macrolides. The *bla*_NDM_ genes were associated with resistance genes against beta-lactams, carbapenems, macrolides, aminoglycosides, sulfonamides, TMP, tetracyclines, rifampin, fluoroquinolones, phenicols, and disinfectants (*qacG* and *qacE*). Similarly, *bla*_OXA-58_-like was observed in co-existence with resistance genes against beta-lactams, carbapenems, macrolides, aminoglycosides, sulfonamides, TMP, tetracyclines, rifampin, fluoroquinolones, phenicols, and disinfectants (*qacEDelta1*, and *qacG*). Moreover, *bla*_OXA-24/40_-like was linked to macrolide resistance genes (**Figure 2**).

### 3.6 The co-occurrence of plasmids and chromosomes harboring carbapenemase genes

**Figure 3:**
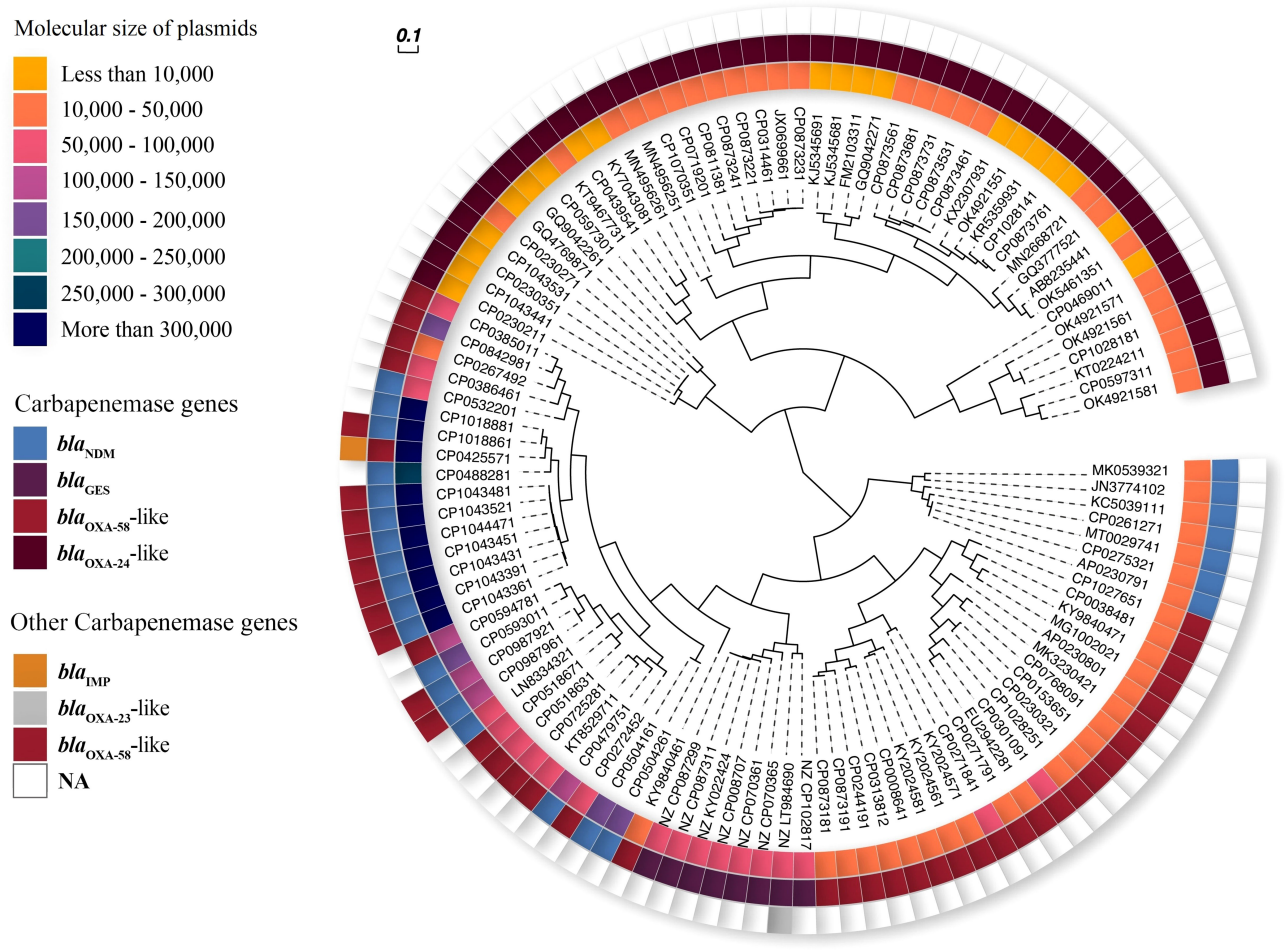
Phylogenetic UPGMA of carbapenemase-harboring plasmids from *A. baumannii.* Inner ring: 8 ranges for size of plasmids (yellow to navy blue color spectrum). Middle ring: carbapenemase genes (blue-*bla*_NDM_, purple-*bla*_GES_, red-*bla*_OXA-58_-like, and crimson-*bla*_OXA-24_-like). Outer ring: other carbapenemase genes (orange-*bla*_IMP_, gray-*bla*_OXA-23_-like, red-*bla*_OXA-58_-like).

Analysis of the data retrieved from the GenBank database revealed the co-occurrence of predominant allele types of carbapenemase genes in the plasmids and chromosomes. This co-occurrence was observed in 12 plasmids, among which 10 harbored both *bla*_NDM-1_ and *bla*_OXA-58_-like genes, one contained both *bla*_OXA-58_-like and *bla*_IMP_ genes, and one harbored both *bla*_GES_ and *bla*_OXA-23_-like genes. Notably, there was no co-occurrence of *bla*_OXA-24_-like and other carbapenemase genes in the plasmids dataset (**Table 1** and **Figure 3**). Furthermore, co-occurrence was found in 19 chromosomes, which can be categorized as follows:17 chromosomes carried both *bla*_NDM-1_ and *bla*_OXA-23_-like genes, one chromosome contained both *bla*_NDM-1_ and *bla*_OXA-58_-like genes, and one chromosome exhibited co-occurrence of *bla*_OXA-23_-like and *bla*_OXA-10_ genes. Notably, there was no co-occurrence of *bla*_KPC-3_ with other carbapenemase genes in the chromosomes (**Table 2**).

### 3.7 The gene repetition in the plasmids and chromosomes

In this study, carbapenemase gene repetitions were identified in seven plasmids harboring *bla*_OXA-24_-like (CP107035.1, CP087324.1, CP087323.1, CP081138.1, CP071920.1, CP031446.1, and JX069966.1), three plasmids harbored *bla*_OXA-58_-like (CP027184.1, CP027179.1, and CP000864.1), and one chromosome (CP091350.1) had *bla*_OXA-58_-like. Carbapenemase gene repetitions were not detected within the plasmids or chromosomes carrying other carbapenemase gene variants.

### 3.8 Genetic environment of carbapenemase genes

**Figure 4:**
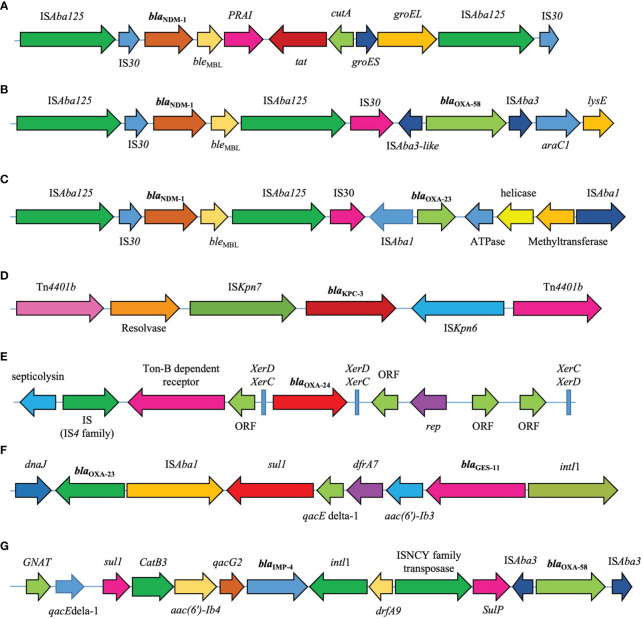
Genetic environments of major carbapenemase genes **(A)** *bla*_NDM-1_ (accession number: KC503911.1). **(B)** *bla*_NDM-1_/*bla*_OXA-58_ (CP104348.1). **(C)** *bla*_NDM-1_/*bla*_OXA-23_ (CP050523.1). **(D)** *bla*_KPC_ (KJ748372.1). **(E)** *bla*_OXA-24/40_-like (CP043954.1). **(F)** The *bla*_OXA-23_/*bla*_GES_ (LT984690.1). **(G)** The *bla*_OXA-58_/*bla*_IMP_ (CP042557.1).

Two copies of IS*Aba125* flanked *bla*_NDM-1_. The *bla*_NDM_ cluster was sequentially embedded in the IS*Aba125*-*IS30*- *bla*_NDM_-*ble*_MBL_-*tat*-*cutA* structure. *bla*_OXA-58_ is located in the IS*Aba3*-*bla*_OXA-58_-IS*Aba3*-like structure. This was followed by *araC1* (a putative transcriptional regulator) and *lysE* (a putative threonine efflux protein). The *bla*_KPC_ gene was flanked by two copies of Tn*4401*. This transposon also harbors the IS*Kpn6*, IS*Kpn7*, transposase, and resolvase genes. The *bla*_GES_ gene is located in a class 1 integron. The *bla*_GES_ gene cassette is downstream of *aacA4* gene, which encodes AAC (6′)-Ib aminoglycoside acetyltransferase. This was followed by the *dfrA7* gene cassette, trimethoprim resistance gene, and *qacEdelta1*. The *bla*_IMP_ gene was found in the *bla*_IMP_-*qacG2*-*aacA4*-*catB3-sul* cassette array and was the most abundant gene in the class 1 integrons. This array contained genes that confer resistance to quaternary ammonium compounds (*qacG*), aminoglycosides (*aacA4*), and chloramphenicol (*catB3*). XerC/XerD-like binding sites flanked the *bla*_OXA-24/40_-like genes. In addition, *bla*_OXA-23_ was flanked by two copies of IS*Aba1.* This arrangement was followed by the ATPase and helicase genes (**Figure 4**).

### 3.9 Comparative analysis of plasmids carrying *bla*_NDM-1_ and *bla*_OXA-58_

**Figure 5:**
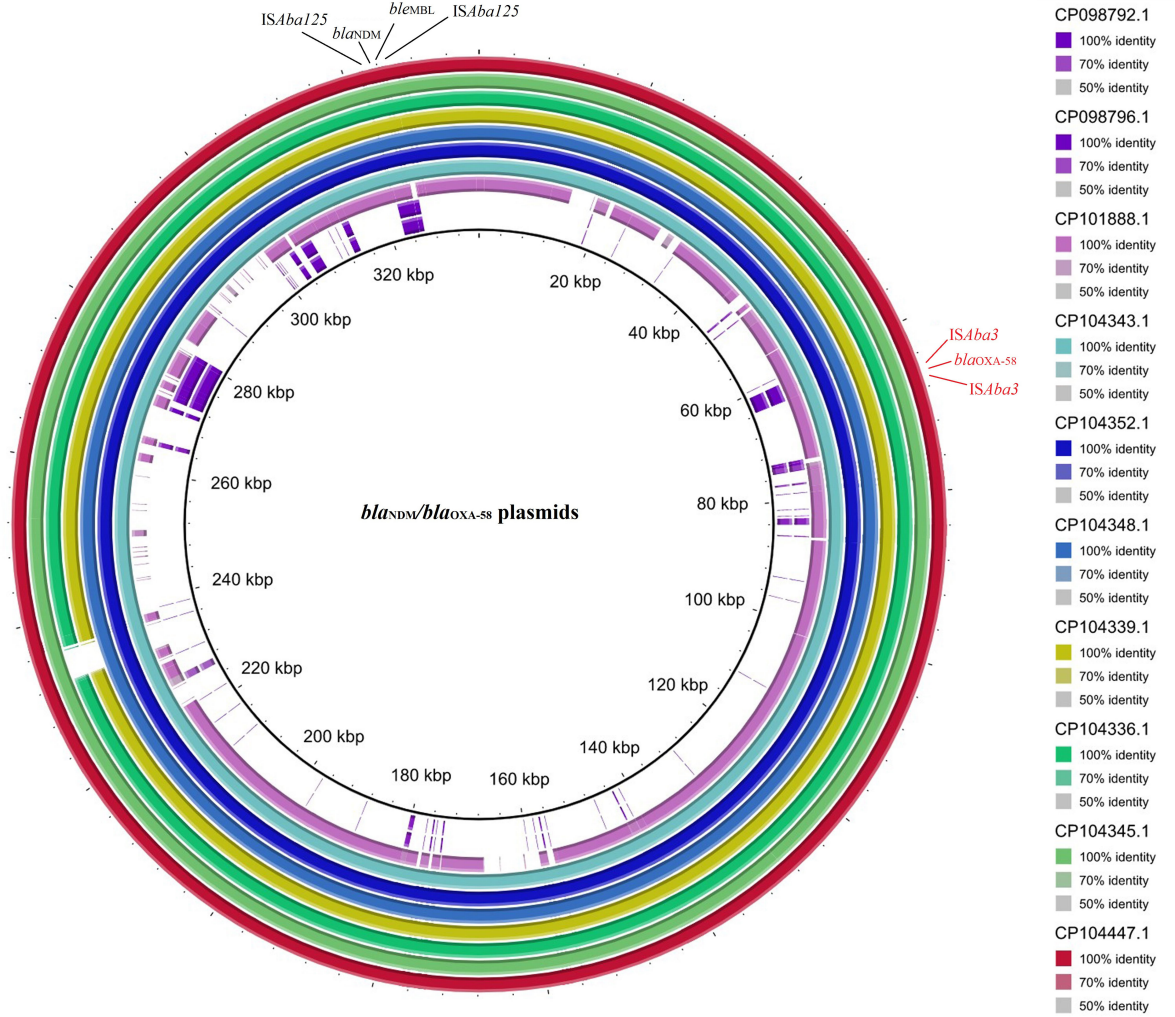
The multiple circular sequence alignment and genetic environment of plasmids harboring *bla*_NDM-1_/*bla*_OXA-58_ in *A. baumannii.* Inner ring: size of plasmids with co-occurrence of *bla*_NDM-1_ and *bla*_OXA-58_, and outer ring: the reference strain. Color spectrum: identity percent of the plasmids with the reference strain.

Multiple circular alignments of the plasmids carrying the co-occurrence of *bla*_NDM-1_ and *bla*_OXA-58_ were performed, including p1VB280820 (CP098792.1), p1VB280821 (CP098796.1), pAC1633-1 (CP059301.1), pABF9692 (CP048828.1), unnamed4 (CP104336.1), unnamed2 (CP104339.1), unnamed2 (CP104348.1), unnamed1 (CP104352.1), unnamed1 (CP104343.1), unnamed2 (CP104345.1), pccbh31258 (CP101888.1), and pCCBH31270 (CP101886.1). In this study, plasmid unnamed2 (CP104447.1) was used as a reference (**Figure 5**). These plasmids appeared to be more homogeneous in their genetic structure, marked by the presence of a set of common genes, including *acrA*, *acrE*, *actP*, *adh*, *aes*, *aphA*, *betI*, *bin3*, *aacA4*, and *acoR*, as well as the antibiotic resistance genes, *bla*_NDM_ **_-_**_1_ and *bla*_OXA-58_, which were consistently present across all plasmids.

Despite their homogeneity, certain regions of the plasmids displayed heterogeneity. For example, plasmid pccbh31258 (CP101888.1), featured unique genes such as *camA* and *cydA*, and plasmid p1VB280820 (CP098792.1), had *rep* and *sasA* genes. Additionally, plasmids unnamed4 (CP104336.1) and unnamed2 (CP104345.1) were identified the unique genes *noc* and *recD2*, respectively.

### 3.10 Clonal relatedness of *A. baumannii* strains carrying carbapenemase genes

**Figure 6:**
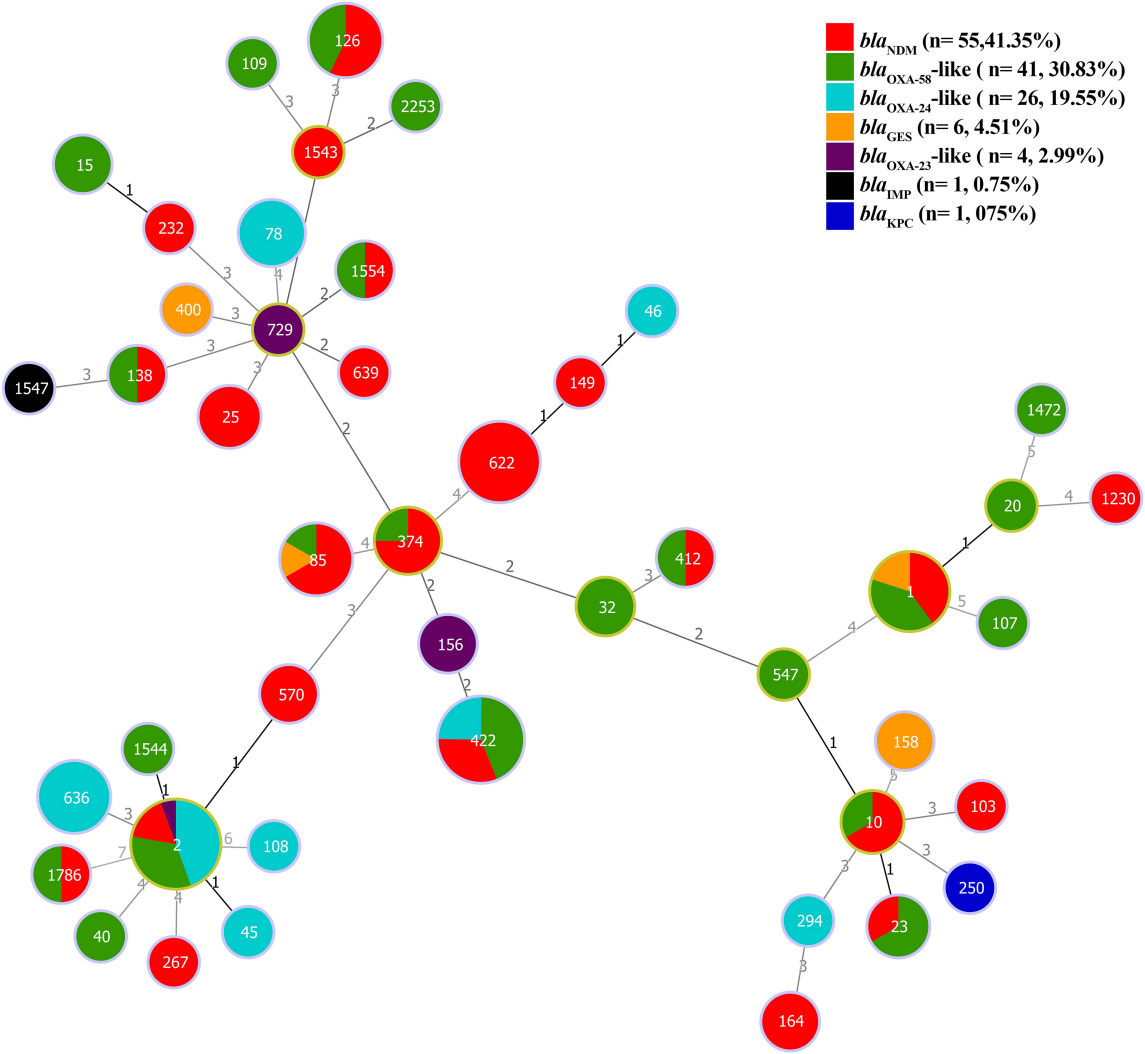
The minimum spanning tree (MST) of STs carrying plasmids containing carbapenemase genes (*bla*_NDM_, *bla*_KPC_, *bla*_IMP_, *bla*_GES_, *bla*_OXA-58_-like, *bla*_OXA-24/40_-like, and *bla*_OXA-23_-like) with a similarity cut-off > 4 allelic types based on multi-locus sequence typing (MLST) scheme. The numbers on the lines indicate allelic differences between the two separate sequence types. Red: *bla*_NDM_, Green: *bla*_OXA-58_-like, Blue: *bla*_OXA-24_-like, Orange: *bla*_GES_, Purple: *bla*_OXA-23_-like, Black: *bla*_IMP_, and Blue Navy: *bla*_KPC_ genes.

The clonal relatedness of *A. baumannii* strains carrying carbapenemase genes can be classified as follows. Most *bla*_OXA-58_-like and *bla*_OXA-24/40_-like genes were associated with ST2^pas^. The *bla*_NDM_ gene were predominantly associated with ST622^pas^. In contrast, the *bla*_IMP_, *bla*_KPC_, and *bla*_OXA-23_-like genes were primarily correlated with ST1547^pas^, ST250^pas^, ST156^pas^, and ST729^pas^, respectively. Some STs were linked to only one specific carbapenemase gene, such as ST1547^pas^ with *bla*_IMP_, ST250^pas^ with *bla*_KPC_, ST156^pas,^or ST729^pas^ with *bla*_OXA-23_-like. Additional STs, including ST2^pas^, ST422^pas^, ST1^pas^, ST85^pas^, ST1786^pas^, ST23^pas^, ST10^pas^, ST412^pas^, ST374^pas^, ST1554^pas^, ST138^pas^, and ST126^pas^, displayed a multi-harboring pattern with carbapenemase genes, including *bla*_NDM_, *bla*_OXA-58_-like, *bla*_OXA-23_-like, *bla*_OXA-24/40_-like, and *bla*_GES_. In a separate analysis of the *A. baumannii* chromosomes, thirty-eight non-redundant chromosomes were identified, revealing 15 different STs. Notably, the co-occurrence of *the bla*_NDM_ and *bla*_OXA-23_-like genes was predominantly associated with ST622^pas^. In contrast, chromosomes carrying *bla*_OXA-58_-like genes were exclusively linked to ST2^pas^. Moreover, chromosomes associated with ST103^pas^, ST85^pas^, and ST250^pas^ carried various carbapenemase genes, including *bla*_NDM-2_, *bla*_NDM-6_, and *bla*_KPC-3_, respectively. Chromosomes harboring *bla*_OXA-23_-like genes were exclusively identified in association with ST156^pas^ (**Figure 6**).

## 4 Discussion

Carbapenem resistant *A. baumannii* poses a significant challenge because it is a leading cause of community-acquired and nosocomial infections, contributing to elevated morbidity and mortality rates (Mirzaei et al., 2020). Carbapenems are last-line antibiotics against these infections. However, MDR and XDR *A. baumannii* isolates complicate treatment (Katip et al., 2022).

The presence of carbapenemase genes, including *bla*_NDM_, *bla*_KPC_, *bla*_VIM_, *bla*_IMP_, *bla*_GES_, *bla*_OXA-58_-like, *bla*_OXA-24/40_-like, and *bla*_OXA-23_-like, could be a public health concern. In addition, carbapenemase genes on conjugative plasmids contribute to higher dissemination rates of these genes between bacteria (Benson et al., 2017). In the current study, bioinformatic tools were used to present more information on the genetic characteristics of *A. baumannii* plasmids and chromosomes harboring carbapenemase genes.

According to the results of this study, *bla*_NDM_ and *bla*_OXA-58_ were the most prevalent carbapenemase genes in the plasmids and chromosomes. Consistent with the current study, Monnheimer et al. showed that the most abundant carbapenemases in *A. baumannii* were belonged to these genes (Monnheimer et al., 2021).

Our study revealed significant findings regarding the co-existence of various aminoglycoside resistance genes, such as *aac(3)-Iid*, *aac(6’)-Ib4*, *aph(3’)-Ia*, *aadA2*, *ant(2’’)-Ia*, *aac(6’)-Ib1*, *aph(6)-Id*, *aph(3’)-Via*, *aph(3’’)-Ib*, *aph(3’’)-Ib*, *aph(6)-Id*, *aac(6’)-Ib9*, *aac(3)-IId*, *aph(3’)-Ia*, *aac(3)-Iic*, *ant(3’’)-Iic*, *aph(3’)-Vib*, *arr-2*, *aph(3’)-VI*, *aac(6’)-Ib9*, and *aac(3)-Iie* with carbapenemases. Furthermore, Nowak et al. (2014) have previously reported similar observations regarding the co-existence of carbapenemases and aminoglycoside resistance genes (Nowak et al., 2014).

Any action to combat the spread of antibiotic resistance genes requires the identification of the potential sources and genetic environment of these genes. The study of MGEs associated with antibiotic resistance genes could provide valuable epidemiological information to identify potential sources. The genetic environment of the carbapenemase genes is consistent with the results of many studies conducted on this topic. Among the MBLs, *bla*_KPC_ and *bla*_NDM_ were associated with Tn*4401*/non-Tn*4401* elements and IS*Aba125*/Tn125, respectively. Whereas, the *bla*_VIM_ and *bla*_IMP_ were associated with class 1 integron (Reyes et al., 2020; Wang et al., 2023). Nguye et al. showed that the *bla*_OXA-58_ is located in an IS*Aba3*-*bla*_OXA-58_-IS*Aba3*-like structure (Nguyen et al., 2020).

In parallel with our study findings, Lasarte-Monterrubio et al. revealed that the *bla*_OXA-24/40_-like was associated with the XerC/XerD-like binding site, while *bla*_OXA-23_ was flanked by IS*Aba* (Lasarte-Monterrubio et al., 2022). Transposons and integrons, found in different plasmids and bacterial clones, are indicative of their transmissibility and mobilization within various genetic elements (Reyes et al., 2020). MGEs, including conjugative plasmids, transposons, integrons, and bacteriophages, act as carriers for the acquisition and transfer of antibiotic resistance genes. They play an important role in transferring resistance genes among bacteria (Nadella et al., 2022). In the current study, the majority of conjugative plasmids were linked to *bla*_NDM_, which may be the primary reason for their high prevalence co-existence with other antibiotic-resistance genes. In the present study, the most prevalent replicon types were R3-T1 and R3-T2. Previous studies have shown that *bla*_NDM-1_ in *A. baumannii* isolates may have chromosomal or plasmid origin (Sánchez-Urtaza et al., 2023). According to the results of current study, Chen et al., demonstrated the co-existence of *bla*_OXA-58_-like with *bla*_NDM_, *bla*_OXA-58_-like with *bla*_IMP_, and *bla*_GES_ with *bla*_OXA-23_-like. This phenomenon is related to the fact that these genes are located on the same conjugated plasmid (Chen et al., 2019). In a recent study, *bla*_OXA-24/40_-like genes were not found to co-occur with other carbapenemase genes. Moreover, CHCs analysis revealed the co-occurrence of *bla*_NDM-1,_ *bla*_OXA-23_, and *bla*_OXA-58_. This finding was consistent with the results of Ramoul et al. (2016). Additionally, we identified the co-occurrence of *bla*_OXA_ *_-_*_565_ and *bla*_OXA-10_.

Carbapenemase gene repetition is a significant evolutionary process that affects environmental adaptation of bacteria. Repetition of antimicrobial genes is a prevalent metabolic factor that gradually changes during evolution, and plays an important role in antimicrobial resistance. Specifically, we found carbapenemase gene repetitions in *bla*_OXA-24_ and *bla*_OXA-58_, which were located on both the plasmids and chromosomes. These duplications might have contributed to the development of carbapenems heteroresistance.

MST results revealed that the predominant STs associated with various carbapenemase genes were ST622^pas^ for *bla*_NDM_, ST2^pas^ for *bla*_OXA-58_-like, and *bla*_OXA-24/40_-like, ST1547^pas^ for *bla*_IMP_, ST250^pas^ for *bla*_KPC_, ST156^pas^ for *bla*_OXA-23_-like, and ST158^pas^ for *bla*_GES_. In addition, there were several other multi-harbor carbapenemase STs, including ST422^pas^, ST1^pas^, ST85^pas^, ST1786^pas^, ST23^pas^, ST10^pas^, ST412^pas^, ST374^pas^, ST1554^pas^, ST138^pas^, and ST126^pas^. These multi-harbor STs were associated with plasmids containing the major carbapenemase genes, including *bla*_NDM_, *bla*_OXA-58_-like, *bla*_OXA-23_-like, *bla*_OXA-24/40_-like, and *bla*_GES_.

In the current study, ST2^Pas^, ST1^Pas^, ST422^Pas^, ST622^Pas^, and ST85^Pas^ were the most prevalent sequence types among the *A. baumannii* isolates. This finding is consistent with the finding of Khuntayaporn et al., study indicated that ST2 is the predominant ST in Thailand (Khuntayaporn et al., 2021). Several studies have consistently shown that the majority of CRAB isolates are associated with the international clone ST2, which has been reported in various studies in Mediterranean countries According to reports from various countries, the clonal diversity of *A. baumannii*, according to the Pasteur scheme of MLST, shows that ST2^pas^, ST1^pas^, and ST3^pas^ are the predominant carbapenem-resistant clones worldwide. In ST studies, ST2 was the most dominant (Abhari et al., 2019; Khuntayaporn et al., 2021). Between 1999 and 2009, a study conducted in four Mediterranean countries (Greece, Italy, Lebanon, and Turkey) revealed that *A. baumannii* outbreaks were predominantly driven by the dissemination of ST2, with fewer contributions to ST1, ST25, ST78, and ST20 (Cherubini et al., 2022). These clones were found to carry *bla*_OXA-58_, *bla*_OXA-23_, and *bla*_OXA-72_ (Nawfal Dagher et al., 2019; Li et al., 2023). In Greece, ST2 is the most common clone circulating in hospitals (Pournaras et al., 2017). In addition, the international clone ST2 is widely distributed in Lebanon (Nawfal Dagher et al., 2019). In a study by Thadtapong et al., ST2 was most frequently identified among colistin- and carbapenem resistant *A. baumannii* isolates. This observation aligns with the findings of the current study, indicating a consistent prevalence of ST2^pas^ in these isolates (Thadtapong et al., 2021). Khorshid et al. investigated the prevalence of various STs associated with genes encoding aminoglycoside-modifying enzymes. Their findings revealed that ST2 was the most prevalent among these STs. Remarkably, ST2, identified as an international clone, exhibits substantial genetic capacity for acquiring antimicrobial resistance genes within its genome (Khurshid et al., 2020).

However, an increased resistance to carbapenems has been reported worldwide. MBLs and CHDL-producing *A. baumannii* isolates, which are responsible for outbreaks, have been reported in different regions worldwide (Reyes et al., 2023). Although OXA-like enzymes weakly hydrolyze carbapenems, they can confer high resistance to carbapenems when associated with IS*Aba1* and IS*Aba125* (Li et al., 2019). MBLs and CHDLs located on MGEs spread rapidly to clonal lineages of *A. baumannii* (Shropshire et al., 2022).

Conjugative plasmids are involved in the rapid spread of CRAB (Chen et al., 2017). Therefore, these strains carrying plasmids and chromosomes harboring different carbapenemases and other antimicrobial resistant genes could pose a major threat to the healthcare system. Therefore, genetic characterization of these plasmids and chromosomes plays an important role in the control of bacteria carrying carbapenemases.

This study provides insights into the genetic structure of plasmids and chromosomes harboring major carbapenemase genes in CRAB. Nevertheless, the study has several limitations. First, the initial dataset and completeness relied on GenBank submissions and annotations. Furthermore, sampling bias exists because the plasmids and isolates studied may not encompass the full spectrum of CRAB strains and carbapenemase gene variations worldwide. In summary, the limitations of this study stem from data source dependence and sampling bias, highlighting the need for caution when interpreting the findings.

## 5 Conclusion

Characterization of the genetic structures revealed that carbapenemase genes appear not only in plasmids but also in the chromosomes of CRAB. The co-existence of plasmids encoding carbapenemases with other antibiotic resistance genes, co-occurrence, and gene repetition of carbapenemases in plasmids and chromosomes were notable findings. Conjugative plasmids containing *bla*_NDM-1_ and *bla*_OXA-58_ pose a threat to the expansion of carbapenem resistance. On the other hand, plasmids harboring *bla*_NDM_ are widespread. *A. baumannii* employ different genetic strategies such as gene repetition and various genetic elements (transposons, integrons, and insertion sequences) to develop efficient resistance against carbapenems. Gene repetition, association of resistance gene cassettes with mobile genetic elements, acquisition of conjugative plasmids, high capacity to acquire carbapenemase genes on plasmids and chromosomes, and expansion of carbapenemases through successful international clones (ST2^Pas^, ST1^Pas^, ST422^Pas^, ST622^Pas^, and ST85^Pas^) play major roles in the development of resistance to carbapenems in *A. baumannii* worldwide. The high consumption of antibiotics in clinical settings exacerbates antimicrobial resistance worldwide. Therefore, a global campaign is necessary to combat against CRAB infection.

## Data availability statement

Publicly available datasets were analyzed in this study. This data can be found here: https://www.ncbi.nlm.nih.gov/genbank/.

## Author contributions

FB: Conceptualization, Data curation, Formal Analysis, Supervision, Writing – review & editing, Project administration, Software, Validation. MB: Data curation, Formal Analysis, Investigation, Methodology, Software, Validation, Visualization, Writing – original draft, Writing – review & editing. HS: Software, Validation, Writing – review & editing. VN: Methodology, Software, Writing – review & editing. MS: Methodology, Software, Validation, Visualization, Writing – review & editing.
